# The assessment of biases in the acoustic discrimination of individuals

**DOI:** 10.1371/journal.pone.0177206

**Published:** 2017-05-09

**Authors:** Pavel Linhart, Martin Šálek

**Affiliations:** 1 Ethology Department, Institute of Animal Science, Praha Uhříněves, Czech Republic; 2 Department of Behvioural Ecology, Faculty of Biology, Adam Mickiewicz University, Poznań, Poland; 3 Institute of Vertebrate Biology, Academy of Sciences of the Czech Republic, Brno, Czech Republic; 4 Faculty of Environmental Sciences, Czech University of Life Sciences Prague, Praha, Czech Republic; Museum National d'Histoire Naturelle, FRANCE

## Abstract

Animal vocalizations contain information about individual identity that could potentially be used for the monitoring of individuals. However, the performance of individual discrimination is subjected to many biases depending on factors such as the amount of identity information, or methods used. These factors need to be taken into account when comparing results of different studies or selecting the most cost-effective solution for a particular species. In this study, we evaluate several biases associated with the discrimination of individuals. On a large sample of little owl male individuals, we assess how discrimination performance changes with methods of call description, an increasing number of individuals, and number of calls per male. Also, we test whether the discrimination performance within the whole population can be reliably estimated from a subsample of individuals in a pre-screening study. Assessment of discrimination performance at the level of the individual and at the level of call led to different conclusions. Hence, studies interested in individual discrimination should optimize methods at the level of individuals. The description of calls by their frequency modulation leads to the best discrimination performance. In agreement with our expectations, discrimination performance decreased with population size. Increasing the number of calls per individual linearly increased the discrimination of individuals (but not the discrimination of calls), likely because it allows distinction between individuals with very similar calls. The available pre-screening index does not allow precise estimation of the population size that could be reliably monitored. Overall, projects applying acoustic monitoring at the individual level in population need to consider limitations regarding the population size that can be reliably monitored and fine-tune their methods according to their needs and limitations.

## Introduction

Monitoring animals is a crucial activity for ecological, behavioural, and conservation science. There is now a growing interest in acoustic monitoring as an alternative or complementary means of monitoring animals [1]. At present, affordable hardware and software products are available making the practical use of acoustic monitoring more accessible [2]. The range of considered acoustic monitoring applications ranges from detection of species presence, number and density of individuals of particular species and their activity in time and space to the assessment of diversity and health of whole ecosystems [1,3,4].

Many studies across the various taxa have demonstrated that, vertebrates universally have individually distinct vocalizations [5–12]. In other words, we can find one or more features in their vocalizations that are less variable within an individual than between individuals. In general, the individual distinctiveness may result from the unique vocal tract anatomy [13] and / or from the presence of unique arbitrary elements or variants in the repertoire of an individual that is used as an “individual signature” [14]. Vocal traits often vary along with physical or behavioural conditions of the individual. On the other hand, true identity signals should remain unaltered along significant time scales [15], There are studies documenting the long-term stability of individual vocal traits in several bird and mammal species [16–18]. Thus, it is possible not only to discriminate between individuals but also to identify them in subsequent time periods [19]. Therefore, individual variation in vocalizations could in principle be used for the detailed and long-term acoustic monitoring of particular individuals.Several studies have shown that the acoustic identification of individuals could be a feasible and valuable tool [20–22], but it is still unclear what are potential biases associated with the particular study methods, design and sampling.

Any recognition task will be limited by the means and principles it uses [23]. The recognition of individuals usually involves two basic steps: 1) extraction of individually distinct features in calls and building a discrimination model and 2) attribution of new call samples to individuals using the discrimination model and evaluation of the discrimination model. While the performance of different classification methods has been compared and discussed before [24,25], the drawbacks and benefits of different methods of feature extraction are less well known. Very often, studies have used measurements of very specific vocalization subunits as individual features [21,26] fine-tuned to a particular species. These measurements may work fine for a single species, but must be developped and tested again and again for each new species. Other studies have used the cross-correlation method, in which the whole spectrogram of a call is compared to spectrograms of the other calls from known individuals and the call is then attributed to the individual with the highest concordance between spectrograms [27]. Cross-correlation does not extract any individual acoustic features per se but practically uses each pixel in the spectrogram as the feature. Cross-correlation scores are based on complete call representations that involve both frequency and amplitude modulation patterns of calls and thus cross-correlation could be probably considered as the most detailed method of call description. Further other studies have focused on more general properties of vocalizations, such as the distribution of the frequency spectrum, the distribution of formants and extracting Mel-frequency cepstral coefficients disregarding the specific composition of call / song subunits [28–30]. Such general approaches might have greater application potential across different species [31]. Few studies evaluated how the detail of the call description might influence the discrimination. An obvious assumption would be that the more detailed the call description the better the discrimination.

In real situations, if discrimination of individuals is used for the monitoring of individuals within a population, the number of monitored individuals would typically be relatively large. Studies investigating individual variation in vocalizations usually involved relatively small numbers of individuals, many of them including less than 20 individuals. Few studies with much larger samples of individuals have show that discrimination success decreases with population size [26,32] which is in accordance with theoretical assumptions [33]. Hence, it is important to understand how the population size being monitored may limit the accuracy of acoustic identification.

The discrimination of individuals is also limited by the quantity of sampling. Studies investigating individual variation typically use 10–20 calls per individual. Such such numbers have been experimentally shown to be sufficient to assess amount of identity information in different species [34]. However, the number of required calls per individual will among other factors depend on external and internal factors affecting call consistency of a particular species. Therefore, more studies on additional species need to be carried out to understand how to scale sampling effort to achieve reasonable discrimination.

When preparing projects on acoustic monitoring of individuals, it might be very helpful to start with a small-scale, pre-screening pilot study to evaluate how much identity information is present and therefore, how many individuals could be discriminated in the species of interest and with the selected call features [34]. Researchers could then go on with a large-scale study if the results of the pre-screening were satisfying. Different measures / indices were used to assess the amount of identity information in vocalizations such as, for example, the score from discriminant analysis [26] or PIC—the potential of individual coding [35]. But only the Beecher’s information criterion H_S_ [33,34] allows conversion to the number of potentially discriminable individuals. However, it is still not well known whether individuality measures, such as H_S_ in particular, are efficient for such pre-screening in different animals.

Owls (Strigiformes), including little owl, are excellent model organisms for acoustic monitoring because they rely on acoustic signals for the long-distance communication and hence are very vocal in different contexts. Moreover, several studies have demonstrated the short-term and long-term stability of individual call characteristics for a variety of owl species which is an important prerequisite for the efficient acoustic monitoring of individuals [6,36,37].

In this study, we assess several factors that could bias results of studies investigating potential for individual discrimination using an extensive sample of targeted recordings of the little owl *Athene noctua* individual males. We simulate effect of different methods and conditions on the discrimination performance at the level of calls and individuals to answer the following questions:

1. What is the difference in the discrimination performance among cross-correlation and two other frequently used methods of call description: call description by the fundamental frequency modulation, and by spectral features of vocalization?
2. How does the number of individuals being monitored affect discrimination performance and individuality index H_S_?
3. Could H_S_ be used to estimate the number of individuals which can be discriminated?
4. How does the number of calls available per male (i.e. sampling effort) affect the number of individual males that can be discriminated in a population?

## Methods

### Ethics statement

The study was done on places with unrestricted public access and on wild animals. Study was purely observational and non-invasive, therefore no special permits were required.

### Study areas and species

The little owl is a non-migratory and sedentary nocturnal predator with stable long-term territories and low dispersal distances (< 15 km) of offspring [38]. We recorded territorial calls of males that function both in territorial defense and mate choice [39,40] and other males can use them to distinguish their neighbours from strangers [41]. The species is strongly associated with open farmlands and its Western and Central European populations have steeply declined over the past 50 years, resulting in highly fragmented distribution and several local population extinctions [42–44].

The study was carried out in two Central European farmlands: 1) northern Bohemia, Czech Republic (50°23'N, 13°40'E) (CZ), 2) eastern Hungary, Hungary (47°33′N, 20°54′E) (HU). The mean population density of the little owl at the CZ site was 0.09 calling males per 10 km^2^ and the population has experienced rapid population decline in recent years [44]. The mean population density of the little owl recorded at the HU site was 5.01 calling males per 10 km^2^ [45] which is one of the highest population densities for this species in Central Europe [38]. The little owls in both study areas bred within the human settlements such as residential buildings and farmsteads [45,46].

### Acoustic recording and analyses

Territorial calls [38] of each male were recorded for three minutes after a short playback provocation (≤ 1 min) inside their territories from up to 50 m distance from the individuals. We used a PMD660 solid-state recorder (sampling frequency 44 100 Hz, no compression) and a Sennheiser ME67 directional microphone to record the calls. Each recording contained calls of one focal male. The recordings were made during comparable, favourable meteorological conditions (without strong wind or precipitation), from sunset until midnight between March and April of 2013–2014. This period covered the mating season. The period and the time of the day for recording were selected with regard to the peak in vocal activity of little owls both within a day and within a season [47]. The recordings were band-pass filtered (500Hz– 2000Hz) and down-sampled to 4000Hz sampling frequency prior to analyses as the fundamental frequency of calls was never bellow 500Hz nor exceeded 2000Hz (the minimum frequency of calls: mean ± SD = 776 ± 98 Hz; the maximum frequency of calls: mean ± SD = 1668 ± 272 Hz). Analyses were done in Avisoft SASLab Pro (Reimund Specht, Berlin). In all cases, spectrograms were generated with following settings: FFT-length was set to 512 points, the Flat Top window function was used, frame size was set to 100%, and window overlap was set to 93.75%.

### Call description methods

**Fig 1:**
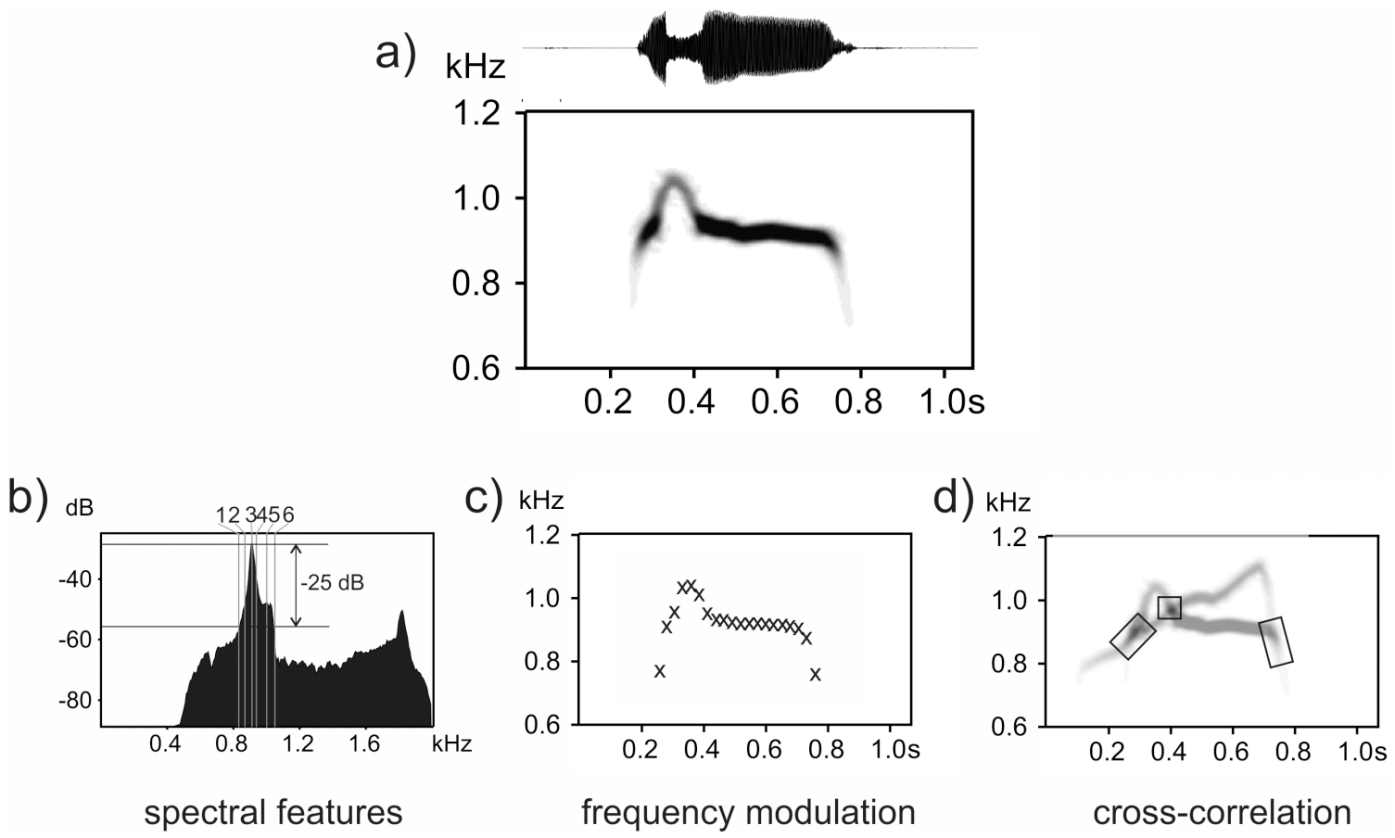
Illustration of little owl call and three methods used for the call description. Example the single territorial call of the little owl male (spectrogram and oscilogram, a), and an illustration of the three call description methods: b) description of call spectral features (1 = minF, 2 = q25, 3 = dF, 4 = q50, 5 = q75, 6 = maxF); c) description of call frequency modulation; and d) cross-correlation of calls (rectangles indicate cross-correlating segments between two displayed calls). Spectrogram settings: FFT-length = 512, window type = Flat Top, window overlap = 93.75%.

We analysed calls from a subset of 54 males for which we had more than 20 calls each (20–41 calls per individual, mean ± SD = 26.9 ± 6.0) with good recording quality (14 individuals came from the CZ population, 40 individuals from the HU population). There were no differences in the spectral features or the frequency modulation of calls between the two populations (spectral features: MANOVA: Wilks = 0.80, *P* = 0.138, frequency modulation: MANOVA: Wilks = 0.88, *P* = 0.882). Hence, we pooled calls from the two populations for all analyses. Territorial calls were described based on the three approaches presented in Fig 1.

The first approach was based on the spectral features of the entire call (Fig 1b). We measured dominant frequency (dF, frequency of highest amplitude on the spectrum), frequencies at the three quartiles of amplitude distribution (q25, q50 and q75, below which lie respectively 25, 50 and 75% of the energy of the call) and minimum and maximum frequencies at -25dB relative to the call peak amplitude (minF, maxF, these two values give approximate range of fundamental frequency). Threshold of -25dB relative to the call peak amplitude was selected for two reasons: 1) setting the threshold makes measurements comparable between calls with variable absolute amplitudes and 2) the specific threshold value was selected based on „try and error” to ensure that it was as close as possible to the minimum and the maximum fundamental frequency of the call but was within the call frequency range in all samples. This approach might be suitable in cases where the modulation of fundamental frequency differs between utterances, for example, in species which do not have a constant number of call elements, in which element types differ in a call sequence, or have noisy calls without clear fundamental frequency. It also can be used in species with complex songs [48]. The same set of features, in general, can be used across different species.

The second approach was based on the description of fundamental frequency modulation (Fig 1c). In this case, we took measurements of fundamental frequency at 20 measuring points (F1 –F20) evenly spaced throughout the duration of calls. Because discrimination based on 20 measuring points was not substantially better (Linhart, unpublished) we mostly used only 10 measuring points so that we took every second measuring point from the original 20 measuring points (see S1 Fig for the representations of F0 modulation and its variation in all 54 males). We used description based on 10 measuring points in all analyses with the exception of the analysis of H_S_ as a predictor of the discrimination performance (see below). The spectral features as well as the modulation of F0 were measured using the ‘Automatic parameter measurement’ tool in Avisoft SAS Lab (Reimund Specht, Berlin). Call duration was also measured in both cases.

In the third case, each call spectrogram was cross-correlated to the spectrograms of all other calls. We used the “Scan for template spectrogram patterns” function in Avisoft SASLab Pro. Settings were: high-pass cutoff frequency = 500Hz; low-pass cutoff frequency = 2000Hz; maximum frequency deviation = 50 Hz. This function returns cross-correlation scores between the template spectrogram and selected files. Each call was successively used as a template and was cross-correlated to all other calls so that we obtained a matrix of cross-correlation scores including all pair-wise combinations of calls in our dataset (Fig 1d).

### Statistical analyses

#### General approach

In cases of spectral features and frequency modulation, we used linear discriminant analysis (LDA) with the leave-one-out cross validation to assign calls to individuals. Discriminant analyses were performed in R using the 'lda' function in the MASS package. We used leave-one-out cross-validation because the results were comparable to those obtained with generally stricter 2-fold cross-validation in the pilot test (see S2 Fig). Prior probability was set equal to each individual (computed as: 1 / number of individuals in a model).

To assign calls to individuals in the case of cross-correlation, we used the matrix of cross-correlation scores for each pair-wise combination of calls. The scores of calls belonging to the same male were then averaged and the call was assigned to the individual with highest average cross-correlation score.

#### Discrimination at the call and individual level

**Fig 2:**
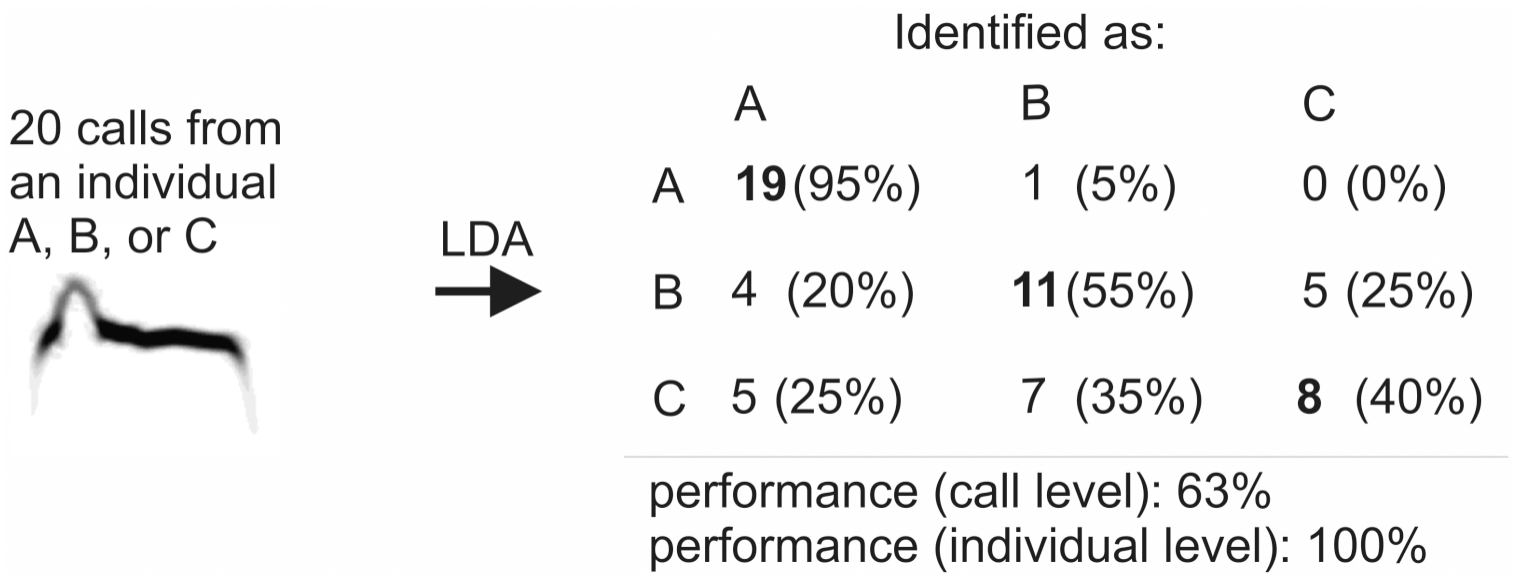
Relationship between the discrimination performance at the call and at the individual level. In this hypothetical example, calling bouts of 20 calls each from individuals A, B, and C are attributed to three individuals by linear discriminant analysis (LDA). Rows represent to which individual calls belonged to and collumns represent to which individual calls were assigned to by LDA. Diagonal represents calls that were attributed to correct individuals. There is 100% discrimination success at the individual level because all three call sets were assigned to correct individual based on majority criterion. Even for C, the set of 20 calls would be correctly identified as belonging to individual C as majority of the calls (40%) were assigned to C. On the other hand, discrimination performance at the call level would be only 63% (overall percentage of correctly assigned calls).

We report discrimination performance at two levels: level of call and level of individual. Similar studies report discrimination performance at the call level only [26,32]; this is equivalent to the frequently reported percentage of calls assigned to the correct individual by LDA. Performance at the level of individual can be easily derived from discrimination performance at the level of calls assuming that the whole set of calls (a calling bout) belongs to a single individual (e.g. when doing targeted recording of a single bird in sight). Althought, some calls from the calling bout might be misatributed to other individuals, majority of the calls should be attributed to the correct individual. Therefore, we attributed the whole set of calls to an individual to whom the most of the calls from the set were assigned to (majority criterion, see Fig 2 for an example). Further, we take 90% of correctly discriminated individuals as a standard for acceptable discrimination at the individual level as this is comparable to the results from visual discrimination based on colour rings [49].

#### Call description method and population size to be monitored

We used custom built R scripts to simulate the effect of increasing population size (increasing number of individuals in LDA) and how it affects discrimination. We started by including calls from two randomly selected males in the LDA. Discrimination performance was evaluated (proportion of correctly identified calls and males). Then in each subsequent step another randomly selected male was added to LDA model and the performance was evaluated until all 54 males were included in the LDA model. In the case of cross-correlation, the procedure was similar but calls were assigned based on cross-correlation scores. The whole run was repeated 20 times to simulate different combinations of individuals. We did not test for a statistical significance of differences in the performance of the methods explicitly. The performance of the three methods was compared graphically using average performance and confidence intervals.

#### Pre-screening of discrimination performance

Beecher’s information statistic H_S_ [33] is a stereotypy index commonly used to estimate the potential of the particular trait to signal individual identity. Higher values of H_S_ indicate greater potential to encode individual identity and are associated with better discrimination in LDA [33,50]. As in case of LDA, H_S_ was computed for sequentially increasing number of randomly chosen individuals from 2–54 males repeated 20 times, but only for the frequency modulation (10 measuring points). H_S_ was computed using the approach and the formulas from the previous studies [33,34]. First, we subjected the original acoustic variables (here F1-F10) to Principal Component Analysis (PCA) [33]. Original acoustic variables were scaled to zero mean and unit variance for PCA. For each of the resulting principle components (PC), we calculated its individual identity information content H_i_:

Eq 1:

$$H_{i}\;=log_{2}\;\sqrt{\frac{F+n-1}{n}}$$ (1)

where *F* is the F-statistic from an ANOVA with the particular PC entered as the dependent variable and the individual as the independent variable, *n* is the number of individual animals in the sample. Significant as well as non-significant F-values were used. The amount of individual identity information in the whole signal H_S_ is subsequently computed simply by summing identity information across all principle components:

Eq 2:

$$H_{s}=\;\sum H_{i}$$ (2)

The estimation of the number of individuals possible to discriminate was computed using another equation used by Pollard et al. [34] that follows from earlier equations used by Beecher [51]:

Eq 3:

$$N=P\;\times \;2^{H_{S}}$$ (3)

where *N* is the number of individual animals distinguishable and *P* is the probability that a target individual’s signature is not held by another individual in the group. For monitoring purposes, we aim for individual traits that will provide a perfect, non-ambiguous identification (*P* = 1). However, this is rarely the case. Precision of the identification is not perfect even when using colour rings, so we set *P* to 0.9 in this study, which is comparable to the colour ring identification [49], a classical method to discriminate individual birds.

We were further interested in whether we could use H_S_ to pre-screen the best combination of acoustic parameters for call discrimination and to estimate the number of males that it would be possible to monitor. Therefore, HS was computed for 23 LDA models that differed in how many and which measuring points (F1 –F20) were included (S1 Table). Each different model represents different amount of identity information available. This has been done for a full set of 54 males H_S_(54) as well as for a subpopulation of 10 individuals H_S_(10) (average from 20 random selections). We used the Spearman’s rank correlation and the linear regression to test associations between: H_S_(10) and H_S_(54), H_S_(10) and the discrimination performance, the number of discriminable males estimated based on H_S_(10) and the number of males discriminated. This has been done to confirm, respectively, that H_S_ computed from the limited subset of males is closely related to H_S_ computed from the full set of males, that greater H_S_ is associated with higher discrimination performance and to see how accurately we can estimate the number of males being discriminated based on H_S_. Linear regression was used when testing for association between the estimated and real number of discriminated individuals. Here, the linear relationship was expected because in an ideal situation, the real number of discriminated individuals should be equal to the estimate.

#### Number of calls and discrimination performance

The effect of an increasing number of calls available for LDA on classification success was also assessed using simulations. This was done again only for the frequency modulation. First we used all 54 males and increased the number of available calls from 2 to 20 to see how this affected discrimination performance. Again, we used 20 repetitions to simulate different call combinations. Finally, we combined both scenarios and simulated the effect of population size and number of calls simultaneously. We used 2 calls per male and increased the number of males from 2–54 (20 repetitions). We noted the average number of males for which the discrimination of individuals dropped under 90%, or where the overall discrimination of calls was lower than 65% (the worst documented call discrimination leading to more than 90% males correctly discriminated in our results), and we took it as a population size that could be reliably monitored for the particular number of calls per male. In subsequent steps this procedure was repeated with an increased number of calls per individual until 20 calls per individual were in the model.

## Results

### Call description method and population size

#### Discrimination performance at the level of calls

**Fig 3:**
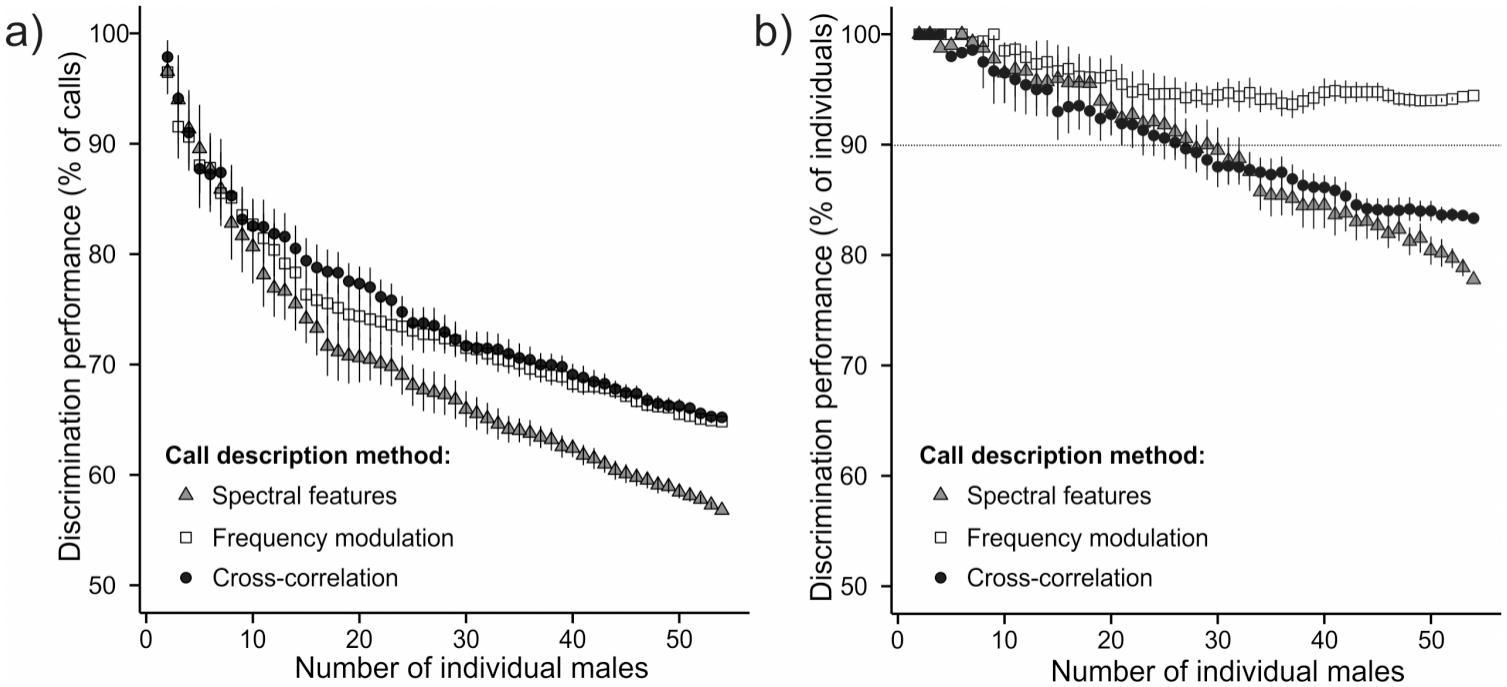
Effect of increasing number of individuals on discrimination performance. Effect of increasing number of individuals on discrimination performance at the level of calls (a) and at the level of individuals (b) for the three call description methods.

Overall, the discrimination performance was high and clearly exceeded discrimination expected by chance (discrimination expected by chance ranged from 1 / 2 = 50% for 2 males; to 1 / 54 = 1.9% for 54 males). The discrimination performance decreased steadily with an increasing number of individuals and ranged from 95% to 57% (Fig 3a). When all 54 males were included, discrimination based on the cross-correlation scores performed best with a 65.2% success rate. However, the performance was similar in the case of LDA based on the frequency modulation (64.8%). LDA based on the spectral features performed the worst (56.8%).

#### Discrimination performance at the level of individuals

When we considered the performance of the three methods in the classification of individuals, we surprisingly found differing results. In this case, discrimination based on the cross-correlation scores (83.3%) performed better than LDA based on the spectral features (77.8%), and the LDA based on the frequency modulation was substantially better than other two methods with a 94.4% classification success (Fig 3b). Interestingly, there was a decrease in the performance for the LDA with the spectral features as well as in cross-correlation, but not in LDA with the frequency modulation which stabilized at about 95% of correctly identified males. On average, it would be possible to monitor 26 males with the cross-correlation, 27 males with the spectral features, but more than 54 males with the frequency modulation with 90% accuracy.

### Pre-screening of discrimination performance

**Fig 4:**
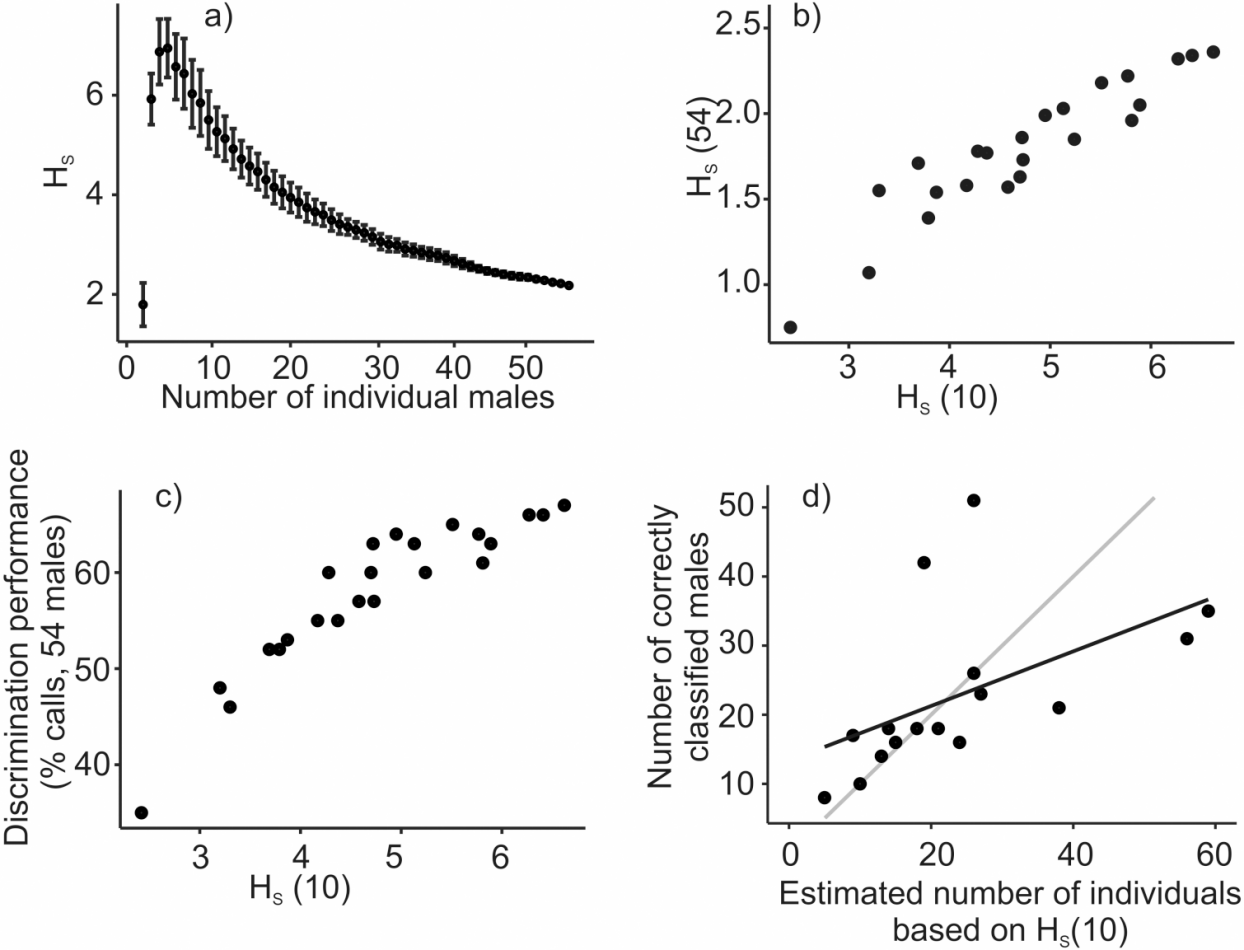
Relationship between the H_S_ and population size to be monitored. (a) H_S_ as a function of the number of individuals in the sample. (b) Relationship between average H_S_ computed from subsample of 10 random individuals H_S_(10) or full sample of 54 males H_S_(54). (c) Relationship between H_S_ and call discrimination performance. (d) Relationship between the estimated and real number of discriminated individuals. Grey line illustrates y = x line for ideal estimates. H_S_ in (b), (c), and (d) was computed for 23 discrimination models that differed in how many and which measuring points (F1 –F20) were included (S1 Table).

H_S_ in our study varied considerably with an increasing number of individuals (Fig 4a). An increasing number of individuals lead first to a steep increase in H_S_; it reached a peak at 5 individuals (H_S_ = 6.94) and then gradually decreased with each additional individual (H_S_ = 2.18 for 54 individuals). These H_S_ values would indicate a very wide and imprecise range of estimates of discriminable individuals—from 4 to 111 individuals could be discriminated assuming a 90% accuracy of recognition depending on how many individuals were sampled to calculate H_S_.

H_S_(10) and H_S_(54) of the 23 models that differed in amount of identity information presented were positively correlated (Spearman rank correlation, R = 0.93, P < 0.001, Fig 4b). This shows, that variable sets having high identity information could be, on average, estimated by using only a subset of 10 individuals despite the fact that the absolute values of H_S_ change substantially with the number of individuals included. Further, Average H_S_(10) values were positively correlated with the performance of call discrimination for all 54 males (Spearman rank correlation, R = 0.93, *P* < 0.001, Fig 4c). Finally, the number of males estimated to be discriminated based on H_S_(10) was significantly positively associated with the number of males correctly discriminated in a full set of 54 males (linear regression: F_1,14_ = 5.32, adjusted R^2^ = 0.22, *P* = 0.037, Fig 4d).

### Number of calls and discrimination performance

**Fig 5:**
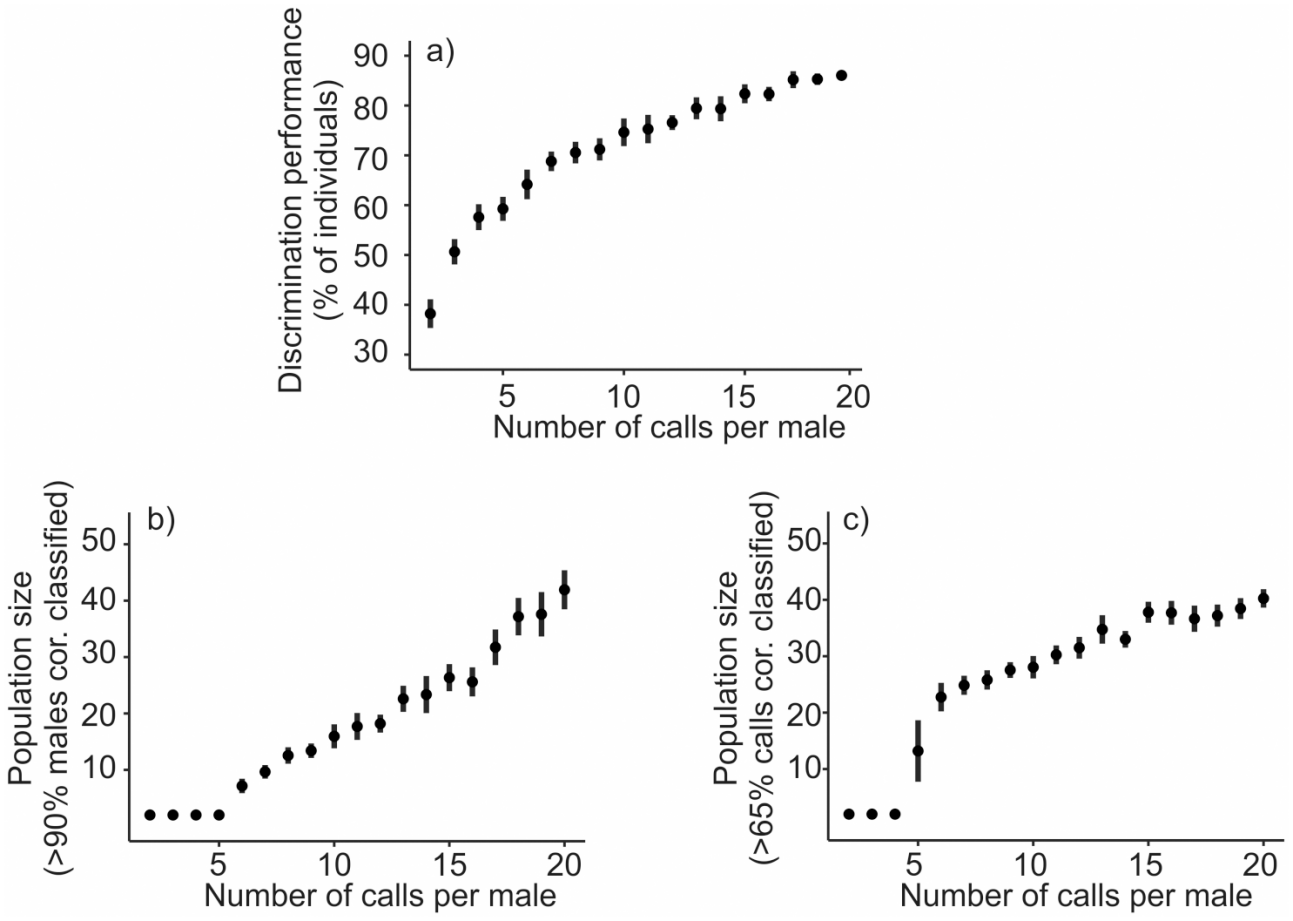
Effect of number of calls per male available on the discrimination performance. (a) Changes in performance with increasing number of calls available for discriminant function (for all 54 males). (b) Population size to be monitored if 90% individuals are to be classified correctly. (c) Population size to be monitored if 65% of calls are to be identified correctly.

The number of calls that were available for building a discriminant function affected call discrimination performance (Fig 5a). Performance increased steeply between 2–9 calls (72% correct at 9 calls) and then continued to increase up to 20 calls per male (90% correct) without reaching a stable plateau.

We further evaluated how an increasing number of calls influences how many individuals can be monitored with 90% precision. With less than 5 calls per individual, the 90% precision was never achieved (Fig 5b). Interestingly, in contrast to call discrimination, from 5–20 calls there was a steady, seemingly linear increase in the size of population that could be reliably monitored with an increasing number of calls per male available. We, therefore, also tried to fix the overall call performance at at least 65% (lowest call discrimination performance documented in our analyses still leading to > 90% of individuals identified correctly) equivalently to fixing the performance at the individual level in the previous analysis (Fig 5c). In this case, there was a huge increase in the number of males from 4 (call discrimination never reached 65% or more) to 6 calls (call discrimination better than 65% for 23 males), a very slow increase with further added calls and no increase at all from c.a. 15 calls per male. This indicates that as few as 6–15 calls might be enough for correct call discrimination, but correct call discrimination is not sufficient for correct individual discrimination which in our case always benefits from adding more calls per individual.

## Discussion

We found that discrimination performance decreased with an increasing number of individual males to be discriminated. Discrimination at the level of calls and at the level of individuals showed substantial discrepancies regarding the choice of the best feature description method and regarding insights into optimum recording effort per male. LDA based on frequency modulation performed best for discrimination of individuals and could be used to monitor more than 54 males if more than 90% males needed to be correctly identified. We found that, contrary to the expectations, the H_S_ individuality index changed profoundly with the number of individuals. Nevertheless, H_S_ correlated well with the call discrimination performance and could be used as relative index of individuality within the studied system. Higher number of calls per male had an important positive effect on discrimination performance. Interestingly, in our case, a high number of calls was not that crucial for discrimination at the level of single calls, but rather for assigning a whole call sequence to an individual. Call inconsistency negatively affected discrimination and was influenced by SNR. Internal factors also seem to cause part of call inconsistency.

### Discrimination at the call and individual level

We show that slight differences at the level of call discrimination may have important consequences for discrimination at the level of individuals (misleading information about performance of the methods, choosing less efficient method, etc.). Researchers should take this into account when selecting the best method for individual recognition. Some studies have used quite strict rules and assigned a call sequence to an individual only if it received more than 50 or even 80 percent hits [22,52]. Our study shows that reliable recognition is possible even with a less strict rule, though at the expense of higher recording effort, i.e. recording more calls per individual.

### Call description method and discrimination performance

We compared the performance of individual recognition based on three different methods. All three methods performed well above chance. Our results should be viewed as optimistic regarding the absolute values of discrimination performance because these might be lower if calls from different calling bouts had been used.

Cross-correlation has been suggested as best performing method for individual recognition [53,54]. In our study, cross-correlation performed slightly better than frequency modulation at the level of calls but fell behind at the level of the individual. Whether this is a general aspect of cross-correlation should be considered in future studies. Both methods, cross-correlation and frequency modulation, outperformed the LDA discrimination based on spectral features. This corresponds to the fact that owl hoots lack pronounced harmonics and formants. Hence, the individual signature is likely to be conveyed by the frequency modulation. Description of the hoot frequency modulation is also commonly used in other studies investigating individual variation of owl hoots [6,55,56].

The three methods differed regarding the call description detail and specificity to the study system. The assumptions on how individuality is encoded in the call differ between the three methods. Cross-correlation might be considered as the most detailed method of call description because every single spectrogram point is considered to compute similarity. On the other hand, spectral features do provide only very general an uncomplete call description. It is, therefore, surprising that the performance of the two methods at the individual level was alike and relatively good: allowing discrimination of c.a. 30 males with 90% accuracy. Probably, good identity signals can be narrowed down to few parameters despite their complexity, so that they can enhance individual recognition and keep low processing demands at the same time [15,57]. To develop acoustic monitoring of individuals, researchers might benefit more from spending time to search for the best marker of identity among different vocalisation types rather than focusing on a single vocalization type.

### Pre-screening of discrimination performance

Because individual discrimination can be compromised in large populations, it is necessary to make use of pre-screening procedures to see whether the species of interest and intended methods will give the appropriate results in cases of large-scale application [34]. Beecher’s informative criterion H_S_; is the only individuality metric currently available that allows a direct conversion of an individuality index into a number of discriminable animals. We found that on average H_S_ computed for a subset of 10 males correlated well with the H_S_ in a complete set of 54 males and even with call classification success. This is in agreement with a previous study [33]. However, the relationship between the actual number of males correctly classified and that estimated from H_S_ was not very tight. Hence, we argue the H_S_ gives a good relative measure of individuality but cannot be used to estimate the size of the population that can be monitored.

Moreover, we found that H_S_ changes very markedly with population size although H_S_, unlike the LDA classification success scores, has been suggested to be independent of sampling [33]. The effect of the number of individuals on H_S_, though small, has also been found previously [34], suggesting that comparisons of H_S_ values from different studies might be problematic. In the original study, there was not apparent effect of number of individuals on H_S_ [33]. Studies might have underestimated this effect due to the numbers of individuals used in previous studies might be drawn from the two sides of the H_S_ peak (Fig 4a). For example, in case we would include 3–10 individuals, we would likely not detect any linear relationship between H_S_ and number of individuals, while including 10–20 individuals into analysis would probably result in negative relationship between the two. Alternatively, the relationship between H_S_ and number of individuals (Fig 4a) does not represent general pattern and could be specific to our study system. Why H_S_ first rises and then falls again and whether it is a general pattern needs to be explained in further studies. But it is possible that the rise reflects the rapid initial expansion of acoustic space each time the new individual is included (i.e. within one dimension the variance between individuals increases while the variance within individuals remains similar).

### Number of calls and discrimination performance

We show that discrimination improves with the number of calls available per individual which is in accordance with a previous study [34]. The previous and this study (Fig 5c) both agree that relatively small number of calls is sufficient to assess the amount of individual information in the calls. However, our study shows that the population size that can be reliably monitored increases approximately linearly with the number of calls available and that acoustic monitoring programs would likely benefit from increased recording effort. Many calls are not crucial at the level of building discrimination function, because the within-individual variation in calls is low. On the other hand, large number of calls becomes neccessary for reliable attribution of those calls to a specific individual if the between-individual variation in calls is not high enough to allow for unambiguous discrimination.

### Conclusions

To conclude, future studies comparing methods of individual discrimination should consider to implement metrics of performance at the level of individuals rather than at the call level only. If researchers plan to individual acoustic monitoring on large scales, they can select the best performing method of call description by pre-screening a limited number of individuals. However, it is not possible to safely estimate the population size for which that method would perform satisfactorily. For small populations, selection of the call description method might not be crucial and even very general methods could be useful. Large scale applications should benefit from colecting large number of calls per individual. Despite the fact that large number of calls per individual is not crucial for building discrimination model, high number of calls per individual is crucial to reliably atribute the sequence of calls to correct individual in larger populations.

An important finding of our study is that discrimination performance (percentage of correctly assigned calls or individuals) and H_S_ are influenced by sampling of the study. Therefore, they should not be directly compared between studies. Robust and accurate pre-screening techniques are currently lacking and should be developed in order to provide a tool to assess the degree of individuality in vocalizations and the efficiency of different methods for the acoustic individual discrimination and identification.

## Supporting information

S1 Dataset Spectral features and frequency modulation for analysed calls. (XLSX)

S2 Dataset Pair-wise cross-correlation scores for analysed calls. (XLSX)

S1 Fig Call spectrograms of 54 individual males. (DOCX)

S2 Fig Comparison of LDA performance with leave-one-out and split sample cross-validation. (DOCX)

S1 Table Overview of 23 different discrimination models based on F1–F20 measuring points. (DOCX)
