# Correction: The assessment of biases in the acoustic discrimination of individuals

**DOI:** 10.1371/journal.pone.0203357

**Published:** 2018-08-29

**Authors:** Pavel Linhart, Martin Šálek

There are errors in the Funding section. The correct funding information is as follows: PL was supported by the Czech Science Foundation (GA14-27925S), Ministry of Agriculture of Czech Republic (MZERO0716), and by the National Science Centre, Poland (under Polonez fellowship reg. number UMO-2015/19/P/NZ8/02507 funded by the European Union’s Horizon 2020 research and innovation programme under the Marie Skłodowska-Curie grant agreement number 665778); MŠ was funded by the Academy of Sciences of the Czech Republic (RVO68081766). The funders had no role in the study design, data collection and analysis, decision to publish, or preparation of the manuscript.
