# Supplementary material for: The assessment of biases in the acoustic discrimination of individuals
Source: PLoS One. 2017 May 9;12(5):e0177206. doi: 10.1371/journal.pone.0177206 (PMC5423633; doi:10.1371/journal.pone.0177206)

**S1 Figure. Call spectrograms from 54 individual males.**

We use modified spectrogram representations (see bellow) that allow to judge the quality and quantity of call variation within and between individuals visually. The input for the spectrograms was the frequency modulation described by 20 measuring points and the duration of the calls. The output was printed using custom build R script. The frequency modulation of each call from an individual was printed in the same picture with partially transparent trace. Thus, if frequency modulation is shared between the calls the trace gets darker. At the same time, differences from the common call modulation pattern (due to mistakes in automatic measuring procedure or due to call variation) are visible and drawn with light traces.


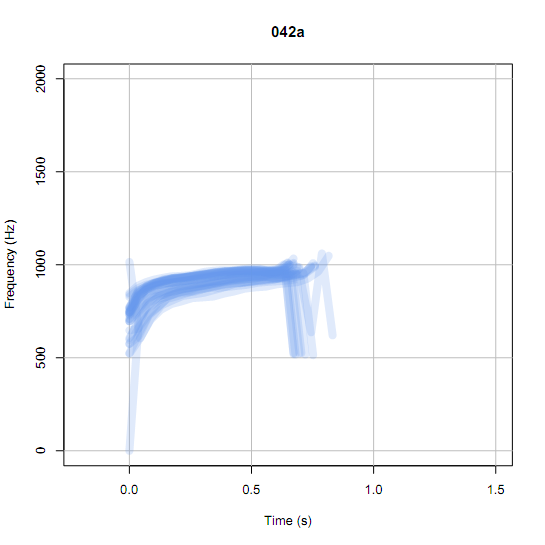


**Hungarian population**

007a 023a 042a 045a


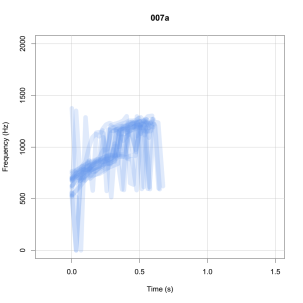

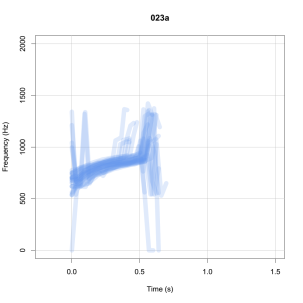

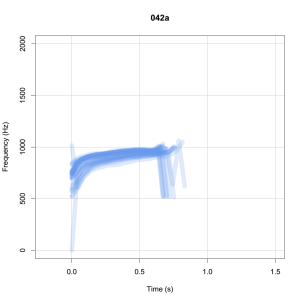

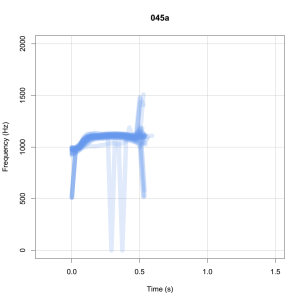


0552 055a 062a 070a


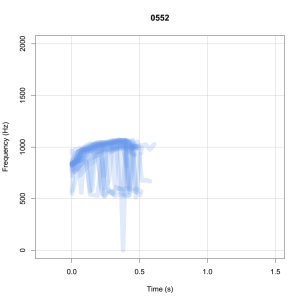

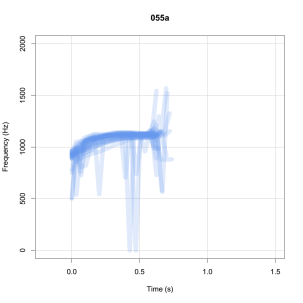

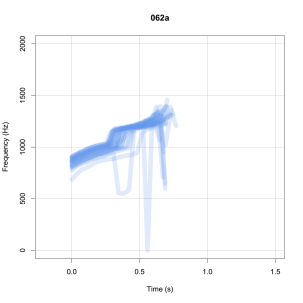

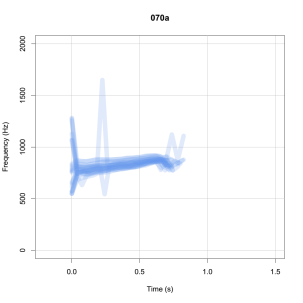


070p 073a 087a 0942


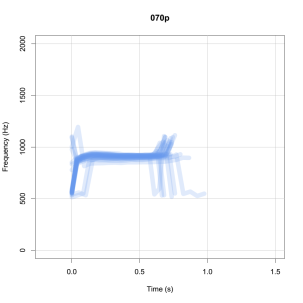

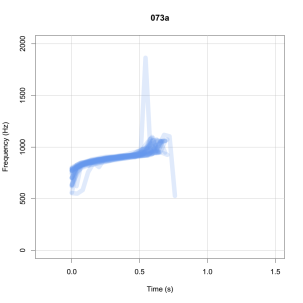

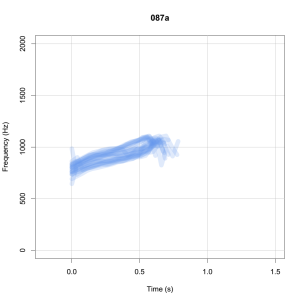

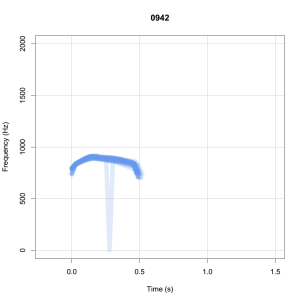


094a 101a 105a 129a


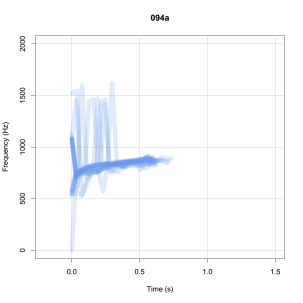

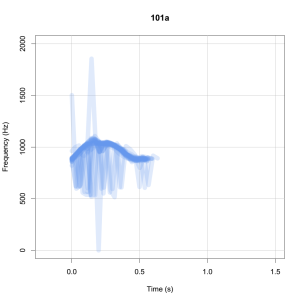

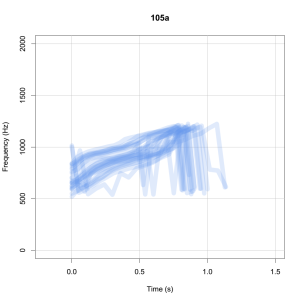

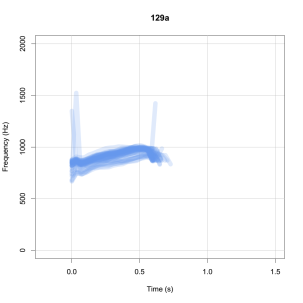


130a 134a 137b 156b


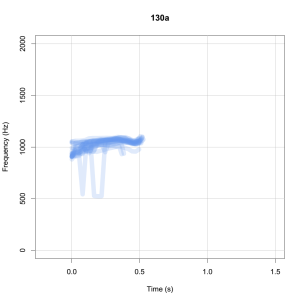

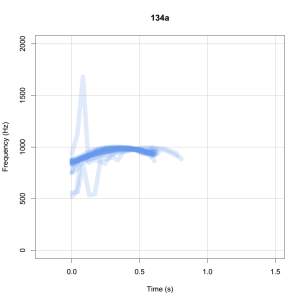

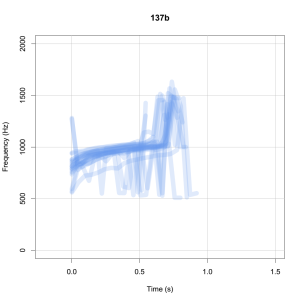

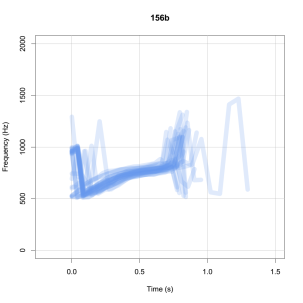


**Hungarian population**

159c 185b 188a 189a


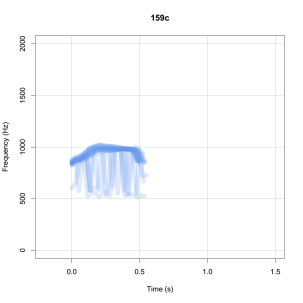

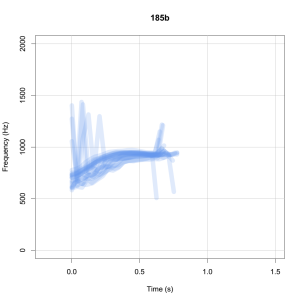

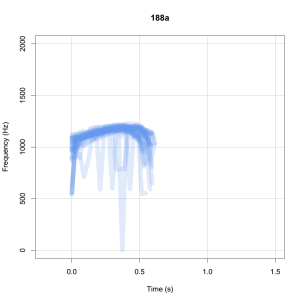

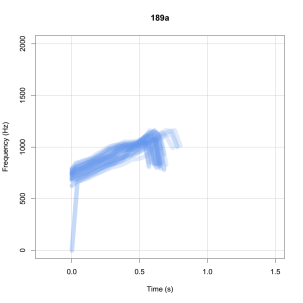


190a 193a 194a 195a


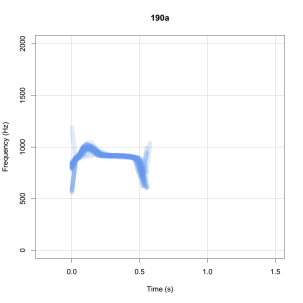

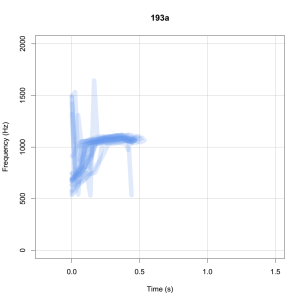

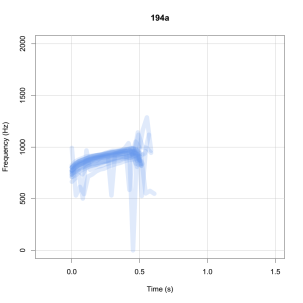

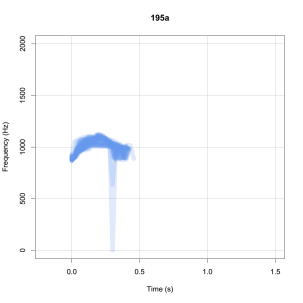


1973 197a 199a 220b


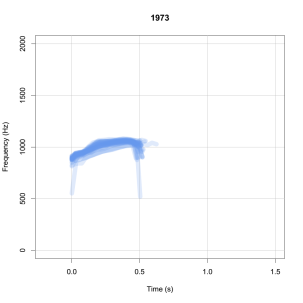

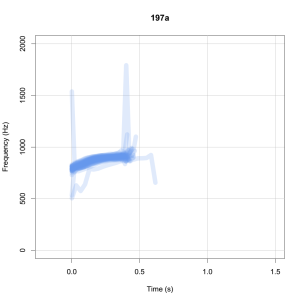

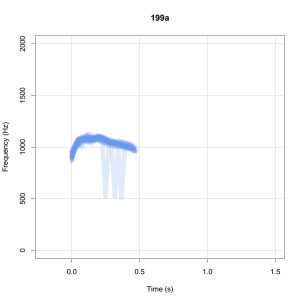

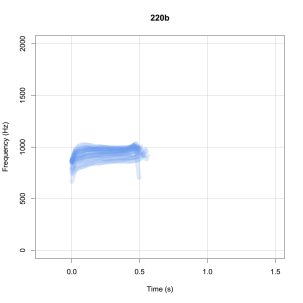


229c 231b 236a 239a


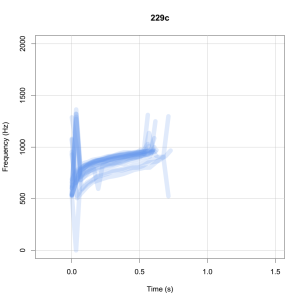

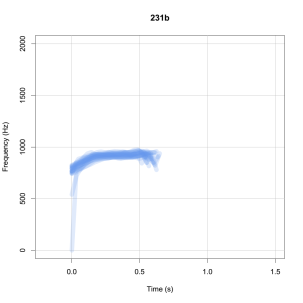

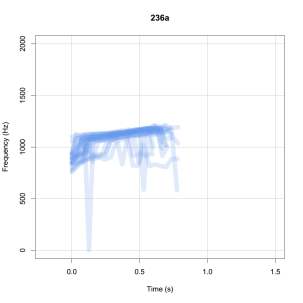

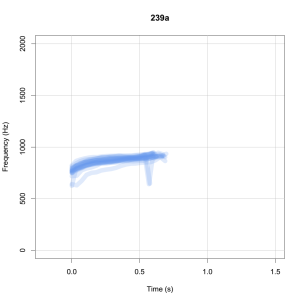


243a 244a 249a breb


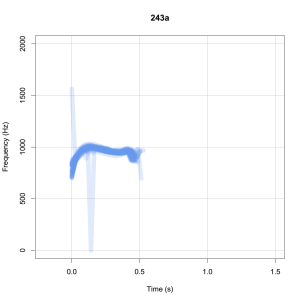

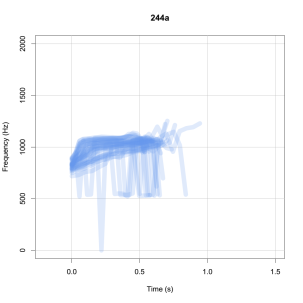

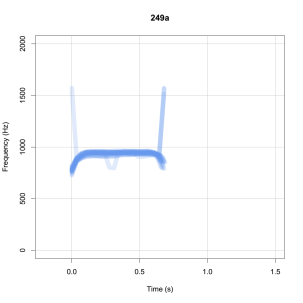

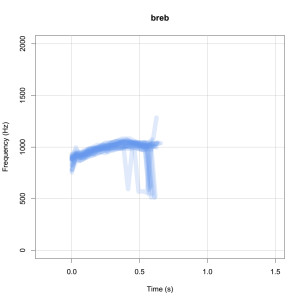


**Czech population**

buda cerb duba kneb


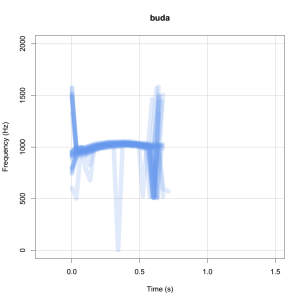

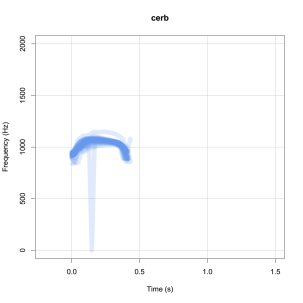

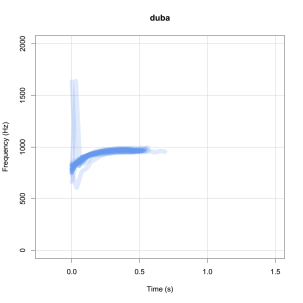

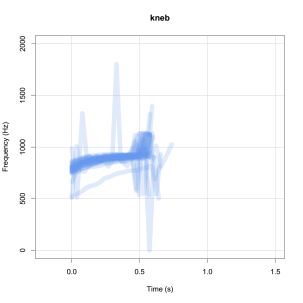


meta pala poda raca


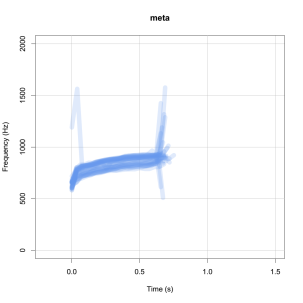

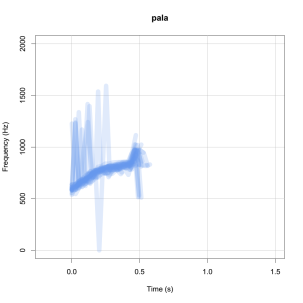

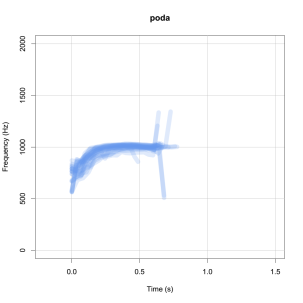

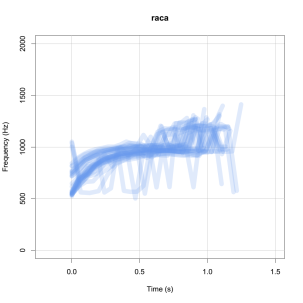


radk skua susa upoa


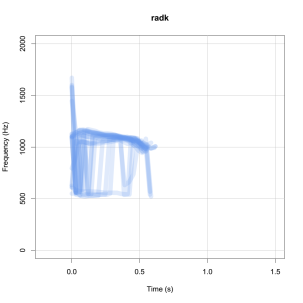

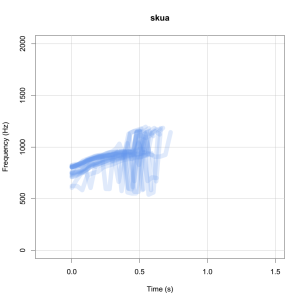

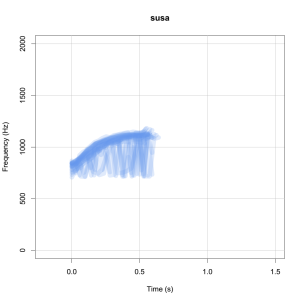

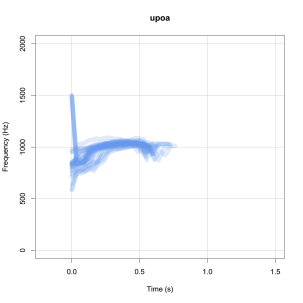


vela vysa


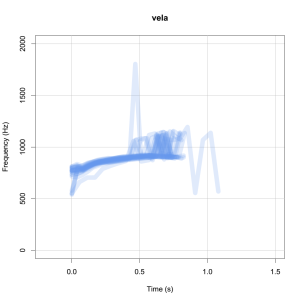

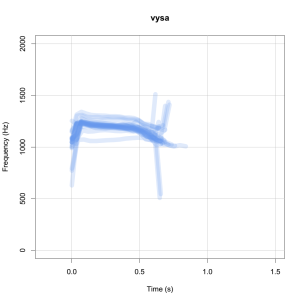

Supplement: S1 Fig — (DOCX) [file pone.0177206.s003.docx]
