# Supplementary material for: The assessment of biases in the acoustic discrimination of individuals
Source: PLoS One. 2017 May 9;12(5):e0177206. doi: 10.1371/journal.pone.0177206 (PMC5423633; doi:10.1371/journal.pone.0177206)
Supplement: S2 Fig — (DOCX) [file pone.0177206.s004.docx]

**S2 Figure. Comparison of LDA performance with leave-one-out and split sample cross-validation.**

Comparison of LDA performance with leave-one-out cross-validation (red) and split sample cross-validation (blue, 2-fold crossvalidation; 10 calls were used to derive discrimination function and other 10 calls were then classified with the discriminant function). We tested the performance of cross-validation methods at the level of call discrimination. We further tested both methods of cross-validation with the spectral features (a) and FM (b). The X-axis indicates number of individuals in the model and Y-axis indicate proportion of calls correctly classified.

a) b)
