# Supplementary material for: The assessment of biases in the acoustic discrimination of individuals
Source: PLoS One. 2017 May 9;12(5):e0177206. doi: 10.1371/journal.pone.0177206 (PMC5423633; doi:10.1371/journal.pone.0177206)
Supplement: S1 Table — (DOCX) [file pone.0177206.s005.docx]

**S1 Table. Overview of 23 different discrimination models based on F1 – F20 measuring points.**

Discrimination performance (proportion of correctly discriminated calls) and Hs was computed from a total number of 23 different discrimination models (see Supplement 3). These models were all based on the description of frequency modulation but differed in the detail of description (2, 3, 5, 10, 15, 20 measuring points) and hence in the precision of available identity information. Four variants were computed for each model with the exception of the full model with 20 measuring points. In one of the models, the measuring points were taken evenly in regular intervals over the whole call, while in the other three variants 2, 3, 5, 10, or 15 measuring points were selected randomly from the all 20 measuring points.

**Model Formula for LDA function**

**Evenly spaced measuring points used**

# shape20 id ~ zdur + zf1 + zf2 + zf3 + zf4 + zf5 + zf6 + zf7 + zf8 + zf9 + zf10 + zf11 + zf12 + zf13 + zf14 + zf15 + zf16 + zf17 + zf18 + zf19 + zf20 (all points)

# shape10 id ~ zdur + zf1 + zf3 + zf5 + zf7 + zf9 + zf11 + zf13 + zf15 + zf17 + zf19 (every second point)

# shape5 id ~ zdur + zf3 + zf7 + zf11 + zf15 + zf19 (every fourth point)

# shape3 id ~ zdur + zf1 + zf11 + zf20 (start, middle and end point)

# shape2 id ~ zdur + zf1 + zf20 (start and end point)

**2, 3, 5, 7, 10 and 15 randomly selected measuring points used (three variants for each number of measuring points)**

# shape2.1 id ~ zdur + zf4 + zf14

# shape2.2 id ~ zdur + zf7 + zf17

# shape2.3 id ~ zdur + zf3 + zf8

# shape3.1 id ~ zdur + zf9 + zf14 + zf20

# shape3.2 id ~ zdur + zf1 + zf10 + zf16

# shape3.3 id ~ zdur + zf2 + zf7 + zf9

# shape5.1 id ~ zdur + zf1 + zf6 + zf7 + zf16 + zf20

# shape5.2 id ~ zdur + zf4 + zf8 + zf11 + zf17 + zf19

# shape5.3 id ~ zdur + zf2 + zf9 + zf10 + zf15 + zf17

# shape7.1 id ~ zdur + zf1 + zf2 + zf3 + zf6 + zf7 + zf12 + zf14

# shape7.2 id ~ zdur + zf3 + zf4 + zf12 + zf15 + zf17 + zf18 + zf20

# shape7.3 id ~ zdur + zf1 + zf7 + zf8 + zf13 + zf14 + zf16 + zf18

# shape10.1 id ~ zdur + zf1 + zf4 + zf5 + zf7 + zf8 + zf10 + zf13 + zf16 + zf17 + zf19

# shape10.2 id ~ zdur + zf4 + zf5 + zf6 + zf8 + zf9 + zf10 + zf13 + zf15 + zf18 + zf19

# shape10.3 id ~ zdur + zf1 + zf3 + zf7 + zf9 + zf10 + zf11 + zf13 + zf15 + zf16 + zf20

# shape15.1 id ~ zdur + zf1 + zf2 + zf4 + zf5 + zf6 + zf7 + zf8 + zf9 + zf10 + zf11 + zf12 + zf13 + zf14 + zf16 + zf18

# shape15.2 id ~ zdur + zf2 + zf4 + zf5 + zf6 + zf7 + zf8 + zf9 + zf10 + zf12 + zf14 + zf15 + zf17 + zf18 + zf19 + zf20

# shape15.3 id ~ zdur + zf3 + zf4 + zf6 + zf7 + zf8 + zf9 + zf10 + zf11 + zf13 + zf14 + zf16 + zf17 + zf18 + zf19 + zf20
